# Neurally Mediated Airway Constriction in Human and Other Species: A Comparative Study Using Precision-Cut Lung Slices (PCLS)

**DOI:** 10.1371/journal.pone.0047344

**Published:** 2012-10-09

**Authors:** Marco Schlepütz, Annette D. Rieg, Sophie Seehase, Jan Spillner, Alberto Perez-Bouza, Till Braunschweig, Thomas Schroeder, Marc Bernau, Verena Lambermont, Christina Schlumbohm, Katherina Sewald, Rüdiger Autschbach, Armin Braun, Boris W. Kramer, Stefan Uhlig, Christian Martin

**Affiliations:** 1 Institute of Pharmacology and Toxicology, RWTH Aachen University, Aachen, Germany; 2 Department of Airway Immunology, Fraunhofer Institute for Toxicology and Experimental Medicine, Hannover, Germany; 3 Encepharm GmbH, Göttingen, Germany; 4 Department of Cardiothoracic and Vascular Surgery, RWTH Aachen University, Aachen, Germany; 5 Institute of Pathology, RWTH Aachen University, Aachen, Germany; 6 Department of Surgery, Luisenhospital Aachen, Aachen, Germany; 7 School of Oncology and Developmental Biology, School of Mental Health and Neuroscience, Maastricht University Medical Center, Maastricht, The Netherlands; Comprehensive Pneumology Center, Germany

## Abstract

The peripheral airway innervation of the lower respiratory tract of mammals is not completely functionally characterized. Recently, we have shown in rats that precision-cut lung slices (PCLS) respond to electric field stimulation (EFS) and provide a useful model to study neural airway responses in distal airways. Since airway responses are known to exhibit considerable species differences, here we examined the neural responses of PCLS prepared from mice, rats, guinea pigs, sheep, marmosets and humans. Peripheral neurons were activated either by EFS or by capsaicin. Bronchoconstriction in response to identical EFS conditions varied between species in magnitude. Frequency response curves did reveal further species-dependent differences of nerve activation in PCLS. Atropine antagonized the EFS-induced bronchoconstriction in human, guinea pig, sheep, rat and marmoset PCLS, showing cholinergic responses. Capsaicin (10 µM) caused bronchoconstriction in human (4 from 7) and guinea pig lungs only, indicating excitatory non-adrenergic non-cholinergic responses (eNANC). However, this effect was notably smaller in human responder (30±7.1%) than in guinea pig (79±5.1%) PCLS. The transient receptor potential (TRP) channel blockers SKF96365 and ruthenium red antagonized airway contractions after exposure to EFS or capsaicin in guinea pigs. In conclusion, the different species show distinct patterns of nerve-mediated bronchoconstriction. In the most common experimental animals, i.e. in mice and rats, these responses differ considerably from those in humans. On the other hand, guinea pig and marmoset monkey mimic human responses well and may thus serve as clinically relevant models to study neural airway responses.

## Introduction

Airway smooth muscle (ASM) contraction is mainly controlled by autonomic neurons innervating the airways and the lung. The autonomic nerves regulate many critical aspects of airway function, e.g. ASM tone, mucus secretion and bronchial microcirculation [1]. The autonomic nerves are subdivided into parasympathetic-cholinergic, sympathetic-adrenergic and non-adrenergic non-cholinergic (NANC) nerves [2].

Several previous studies indicate that the innervation of the lung differs considerably between and within species. For instance, the relative tissue content of the neuropeptides substance P and calcitonin gene related peptide (CGRP) is much higher in guinea pigs than in rabbits or marmosets [3]. Concerning acetylcholine, rat tracheas store about tenfold more of the neurotransmitter than guinea pig trachea or human bronchi [4]. Neurotransmission can also be modulated differently, e.g. cholinergic neurotransmission is facilitated by neurokinin receptors in rabbit (NK1- and NK2-receptor) and guinea pig (NK1 receptor) airways, whereas those receptors have no effect in human airways [5]. Within one species, the same neuropeptides can have opposing effects. Substance P or neurokinin A cause bronchorelaxation in tracheal preparations from Sprague-Dawley and Wistar rats, but contract those from Fischer 344 rats [6]–[9]. Consequently, lung innervation is heterogeneous and extrapolation of pharmacological interventions from laboratory animals to the human situation is difficult. Given all these problems it appears important to identify an experimental animal model that reflects the human situation.

An established *ex vivo* model to study bronchoconstriction in different species is the technique of precision-cut lung slices (PCLS). PCLS were initially introduced for the study of airway pharmacology in rats [10]. Since then this method has been adopted for other species such as mice [11], [12], guinea pigs [13], horses [14], sheep [15], non-human primates [16] and humans [13], [17], [18]. PCLS are highly useful in pharmacological [13], [18]–[22] and toxicological studies [12], [23], [24]. PCLS, that preserve the respiratory tract both morphologically and functionally, have several advantages: First, airway contractions are auxotonic since airways are embedded in their surrounding tissue [11]. Second, contractions of vessels and airways can be distinguished [25]. Third, many slices are obtained from one subject, thereby reducing the number of animals and providing the opportunity of an internal control. Fourth, small and large airways are accessible [10], [21], [22]. Using this approach we showed that with respect to bronchoconstriction small airways compared to large airways are more sensitive to methacholine, serotonin, the thromboxane prostanoid receptor agonist U46619 and allergens in lungs of different species [18], [22], [26]. On the other hand, at least in rats, small airways are less sensitive to EFS than large airways [21].

The present study was designed to systematically study the neuronally mediated airway constriction in different mammals. The use of identical experimental conditions permitted a high degree of comparability among the species studied: mouse, rat, guinea pig, sheep, marmoset and human. Our findings show remarkable differences between all species and suggest that guinea pig PCLS are the best approach to study neuronal mechanisms of airway tone regulation relevant to humans based on the current results.

## Methods

### Animals and human material/Ethics statement

Harvesting of lungs from laboratory animals was approved by the Landesamt für Natur, Umwelt und Verbraucherschutz Nordrhein-Westfalen (LANUV, approval-ID: 8.87–51.05.20.10.245). Balb/c mice (21–23 g) were obtained from Harlan Winkelmann (Borchen, Germany), Wistar rats (200–300 g) from Janvier (Le Genest St. Isle, France) and Dunkin Hartley guinea pigs (340–550 g) from Charles River (Sulzfeld, Germany). Lungs from adult marmoset monkeys (*Callithrix jacchus*) were obtained from the German Primate Center (Göttingen, Germany). Sheep lungs were taken from adult Texel sheep kept at the Maastricht University Medical Center (Maastricht, Netherlands). Care and housing conditions of the animals complied with the regulations of the European Parliament and the Council Directive on the protection of animals used for scientific purposes (2010/63/EU) and the National Institutes of Health Guide for the Care and Use of Laboratory Animals. Human PCLS were prepared from patients undergoing lobectomy due to cancer. After pathological inspection, cancer free tissue from tumor-far parts of the lobes was used. The experiments were approved by the Ethik-Kommission an der medizinischen Fakultät der Rheinisch-Westfälischen Technischen Hochschule Aachen (RWTH Aachen) and all patients gave written consent.

### Chemicals

Atropine, capsaicin, magnesium sulphate, methacholine, neostigmine, Ruthenium red, SKF96365 (1-(β-[3-(4-methoxyphenyl)propoxy]-4-methoxyphenethyl)-1H-imidazole) and standard laboratory chemicals were purchased from Sigma (Steinheim, Germany). Substances for cell culture were obtained from PAA laboratories (Cölbe, Germany).

### PCLS

PCLS were prepared as previously described [22] and used one day after preparation. Briefly, whole lungs (mouse, rat, guinea pig, marmoset) or lung lobes (sheep, human) were filled via the trachea or main bronchus, respectively, with 1.5% (w/v) low-melting agarose and cooled on ice to harden. Tissue cores (10 mm in diameter) with a penetrating airway in its center were prepared by a rotating sharpened metal tube and PCLS were cut perpendicular to the airway by means of a Krumdieck tissue slicer (Alabama Research and Development, Munford, Al, USA) filled up with slicing medium (2 mM CaCl_2_, 1 mM MgSO_4_, 5 mM KCl, 116 mM NaCl, 1 mM NaH_2_PO_4_, 17 mM glucose, 26 mM NaHCO_3_, 25 mM HEPES). As the lungs from mice were too small for tissue coring, the left and right lung were separately embedded in 3% (w/v) agarose before cutting. PCLS were harvested and taken into culture (37°C, 5% CO_2_ atmosphere). The incubation medium was the same as the slicing medium, except for the additional supplementation of 1 mM sodium pyruvate, 2 mM glutamine, amino acids (PAA Laboratories, M11-002, 1∶50) and vitamins (PAA Laboratories, N11-002, 1∶100). For measurements, only slices with airways free of agarose, with beating cilia and an intact and relaxed ASM layer were used.

### Electric field stimulation (EFS)

EFS were performed as described before for rats [21]. Briefly, a PCLS was transferred to a cavity of a standard 12-well plate, placed between two platinum electrodes, weighted down by a Teflon ring and 1 mL incubation medium was added. The electric field was delivered by a Hugo Sachs Electronics Stimulator II (March-Hugstetten, Germany). A standard electric stimulation comprised of a stimulation train lasting 3.3 min with a train rhythm of TR = 60 s, a train width of TW = 2.5 s, a frequency of F = 50 Hz, a pulse duration of B = 1 ms and a current of A = 200 mA (equal to a voltage of 40 V). Airways were monitored by videomicroscopy and used for quantification. Images were analyzed using Optimas 6.5 software (Optimas, Bothell, WA, USA). The airway area before the first stimulation was defined as 100% initial airway area (IAA).

### Frequency response curve

In addition to the standard EFS protocol, EFS was conducted at increasing frequencies (0.4–100 Hz). Each frequency was applied once for 2.5 s and after a pause of one minute the next frequency was examined.

### Pharmacological interventions

Neural stimulation was verified by the use of high magnesium concentrations known to block neuromuscular transmission [27], [28]. PCLS were repeatedly stimulated. The first stimulation train was carried out in the absence of magnesium sulphate. The second stimulation followed 30 min after the addition of 10 mM magnesium sulphate. Subsequent addition of 10^−4^ M methacholine confirmed normal ASM physiology.

Functional innervation was characterized by the use of pharmacological compounds. 10 µM neostigmine and/or 10 µM atropine, concentrations which have been shown to be effective before [21], were added before a particular EFS train was used to study parasympathetic cholinergic innervation. Exogenous addition of 10 µM capsaicin, a compromise according to two former studies [3], [29], was used to study eNANC innervation. The influence of TRP channels on eNANC responses of guinea pig PCLS was examined by the use of 10 µM ruthenium red or 30 µM SKF96365 [30].

### Statistics

Non-linear regression and statistical analyses were performed using GraphPad Prism 5 (GraphPad Software, Inc., La Jolla, USA) or JMP 9 (SAS Institute, Cary, NC, USA). Homoscedasticity was checked by the Bartlett test. If variances were unequal, unpaired data were either compared by the Mann-Whitney test (2 groups) or the Steel-Dwass test (>2 groups); if variances were equal by the t-test (2 groups) or analysis of variance (ANOVA, >2 groups) followed by the Tukey test. Paired data were analyzed by the paired t-test. The statistical test used is indicated in the legends of the table and figures. The median effective frequencies (EF_50_) were calculated by four parameter logistic regression and compared by the F-test. P<0.05 was always considered significant.

## Results

**Figure 1:**
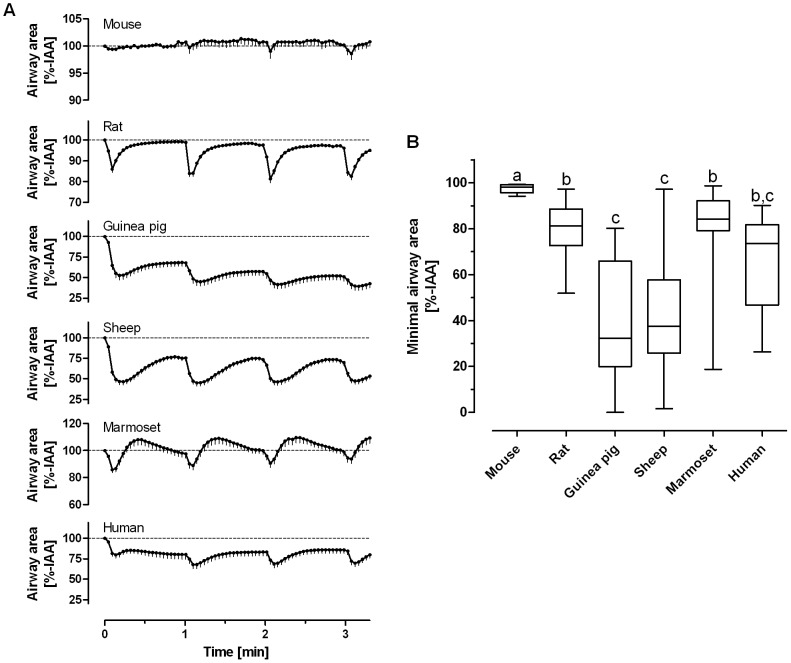
Airway response of precision-cut lung slices (PCLS) from distinct species to electric field stimulation (EFS). PCLS from indicated species were stimulated repeatedly at F = 50 Hz, B = 1 ms, A = 200 mA, TW = 2.5 s, TR = 60 s for 3.3 min, i.e. each PCLS received 4×EFS. **A.** The time course of airway area in successive EFS trains is depicted as mean - SEM for each species; please note the different scales **B.** The statistical evaluation based on the minimal airway area is shown as Box and Whiskers plot. Groups not indicated by the same letter are significantly different at p<0.05 according to the Steel-Dwass comparison test; of note only the mouse was statistically different from human; (species/number of PCLS/number of subjects: mouse/7/5, rat/46/16, guinea pig/25/5, sheep/39/8, marmoset/32/5, human/17/5)

**Figure 2:**
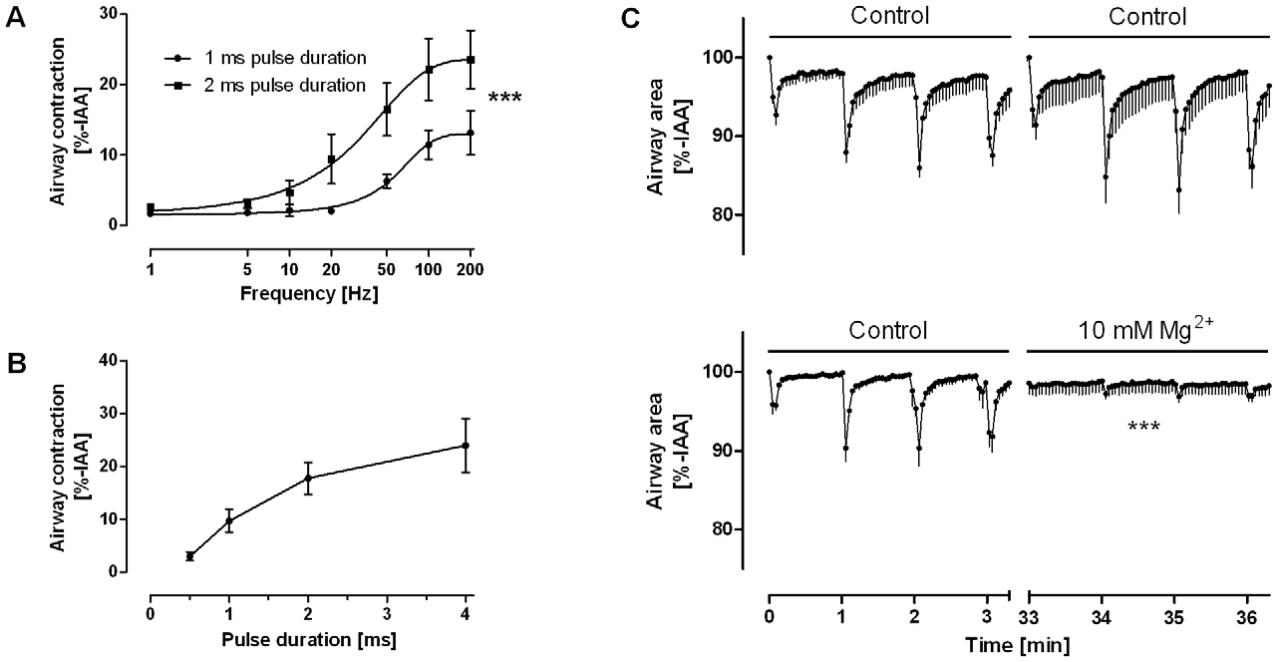
Electrically activated airway contractions in mouse PCLS require higher electric fields and are neurally mediated. A. Frequency response curve: The frequency (F) was increased from 1 Hz to 200 Hz, while the other EFS settings were kept constant at B = 1 ms/2 ms, A = 200 mA≙40 V and TW = 2.5 s; data are shown as mean±SEM (1 ms: n = 5 PCLS from 5 mice; 2 ms: n = 6 PCLS from 6 mice). Frequency response curves were calculated by four parameter logistic regression and compared by the F-test; ***, p<0.001. B. Pulse duration was increased in EFS of murine PCLS from 0.5 ms to 4 ms, while the other parameters were kept constant at F = 50 Hz, A = 200 mA≙40 V and TW = 2.5 s; data are shown as mean±SEM (n = 6 PCLS from 6 mice). C. PCLS from mice were stimulated repeatedly at F = 50 Hz, B = 2 ms A = 200 mA and TW = 2.5 s each minute for 3.3 min. Upper panel: Control stimulations, each EFS train was conducted without pharmacological interference. Lower panel: 10 mM MgSO_4_ was added 30 min prior to the second EFS train. Magnesium was able to block airway responses indicating specific neurally-induced bronchoconstriction. Data are shown as mean±SEM (n = 5 PCLS from 5 mice); ***, p<0.001 in the t-test on the minimal airway area before and after the application of magnesium.

Airway responses to EFS differed largely between species. Airways in PCLS from guinea pigs, sheep and humans contracted at maximum by about 40–60% and did not revert to the initial area during the one minute interval before the next electric impulse was applied (figure 1A,B). However, airways in sheep PCLS revert completely, if recovery phase between stimulations is prolonged (data not shown). PCLS from rats and marmoset contracted reversibly by about 20% (figure 1A,B). Marmoset airways also showed a unique behaviour in that contraction was followed by relaxation that exceeded the original airway caliber (IAA 116±18%; figure 1A). Airways from mice did not respond in the range of the EFS conditions studied here. Since the mouse is such a common laboratory animal, we examined whether neural activation is possible at all in mouse PCLS by using harsher EFS conditions. In fact, the application of substantially higher frequencies or pulse durations led to airway contractions also in mouse PCLS (figure 2A,B). These were blocked by magnesium indicating that the responses were still of neural origin even at these harsher conditions (figure 2C).

**Figure 3:**
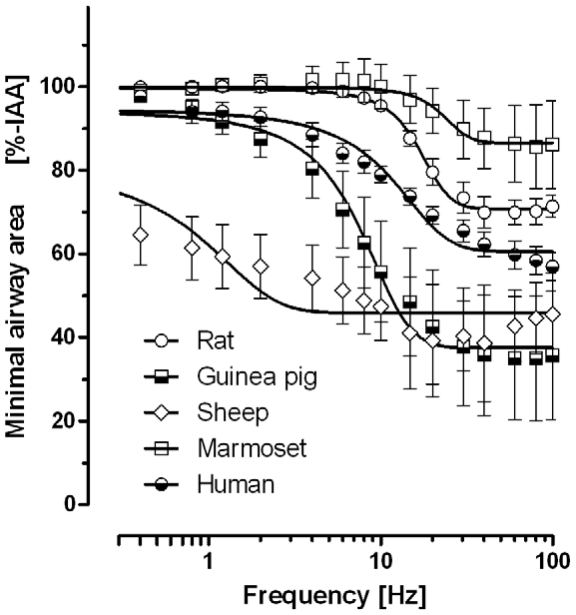
Frequency dependence across species. Frequency response curves were performed on PCLS from the species indicated. Except for the frequency (F), which was increased from 0.4 Hz to 100 Hz after a single train at the distinct frequency each minute (TR = 60 s), EFS settings were kept constant at B = 1 ms, A = 200 mA and TW = 2.5 s. Sigmoidal curves, in which the top was constrained to 100% of the initial airway area [IAA], were plotted and the half-maximal response frequencies (EF_50_±SEM) were determined: rat 16.5±0.3 Hz, guinea pig 7.0±0.5 Hz, sheep 0.4±0.1 Hz, marmoset 22.8±3.6 Hz, human 10.2±0.4 Hz. The four parameters in logistic regression were compared by the F-test and except for the guinea pig (p = 0.23), EF_50_ values from all species (rats: p<0.001; sheep: p<0.01; marmoset (p<0.05) were significantly different from the human EF_50_. Data are shown as mean±SEM (species/number of PCLS/number of subjects: rat/7/6, guinea pig/5/5, sheep/9/9, marmoset/4/3 and human/5/5).

The mouse example demonstrated how species-dependent neuronal excitability may be. Therefore, to further compare the neuronal excitability of the other species, frequency response curves were conducted. Analyzing the frequency response curves for the half-maximal response (EF_50_±SEM) (figure 3) revealed the following order of species sensitivity to EFS: sheep (0.4±0.1 Hz)>guinea pig (7.0±0.5 Hz) = human (10.2±0.4 Hz)>rat (16.5±0.3 Hz). The EF_50_ values for humans and guinea pigs did not differ statistically. The EF_50_ of marmoset was 22.8±3.6 Hz, but the frequency response curve neglects the relaxant response and therefore the EF_50_ appears higher than without relaxation.

To show that the EFS-induced airway contractions were of neural origin, we repeated the experiments in the presence of magnesium that competes with neural calcium channels (N-type calcium channels) and thus prevents neural responses. On the other hand, magnesium does not act directly on smooth muscle cells [27], [28]. Accordingly, EFS performed in the presence of 10 mM magnesium inhibited airway reactions in all species, but did not alter responses to exogenously added methacholine (table 1) demonstrating that the EFS-induced responses depended on neural transmission in all species.

**Table 1:** EFS-induced airway contractions are neurally mediated.

Species	EFS		Methacholine	
	Change in airway area [%-IAA]		Minimal airway area [%-IAA]	
	w/o Mg^2+^	10 mM Mg^2+^	w/o Mg^2+^	10 mM Mg^2+^
Rat	19.0±3.9	1.4±0.3 **	13.5±6.7	30.0±5.2
Guinea Pig	44.3±18.8	3.9±2.1 *	0.0±0.0	7.3±4.2
Sheep	54.4±11.3	7.6±4.1 ^††^	10.1±5.4	18.4±10.0
Marmoset	46.0±8.5	13.2±3.7 ^††^	39.0±10.3	38.2±6.6
(Con./Rel.)	(28±11/18±8)	(11±4.5/2±1.4)		
Human	33.6±9.0	17.4±7.1 ^§§^	34.0±9.2	31.4±7.8

Magnesium competes with calcium at the terminal synapse and prevents the release of neurotransmitters resulting in a neuromuscular block [27], [28]. The muscarinic receptor agonist methacholine was used to confirm that Mg^2+^ did not affect airway smooth muscle directly. Con., contraction in marmoset PCLS, i.e. deviation below baseline airway area before EFS; EFS; electric field stimulation; IAA, initial airway area; Mg^2+^, magnesium; Rel., relaxation in marmoset, i.e. deviation above baseline airway area before EFS; w/o, without; data are shown as mean±SEM (species/number of PCLS/number of subjects: rat/5/5, guinea pig/4/4, sheep/4/4, marmoset/6/5, human/6 EFS, 5 methacholine/6 EFS, 5 methacholine); *, p<0.05; **, p<0.01 in Mann-Whitney test; ^††^, p<0.01 in t-test; ^§§^, p<0.01 in paired t-test.

**Figure 4:**
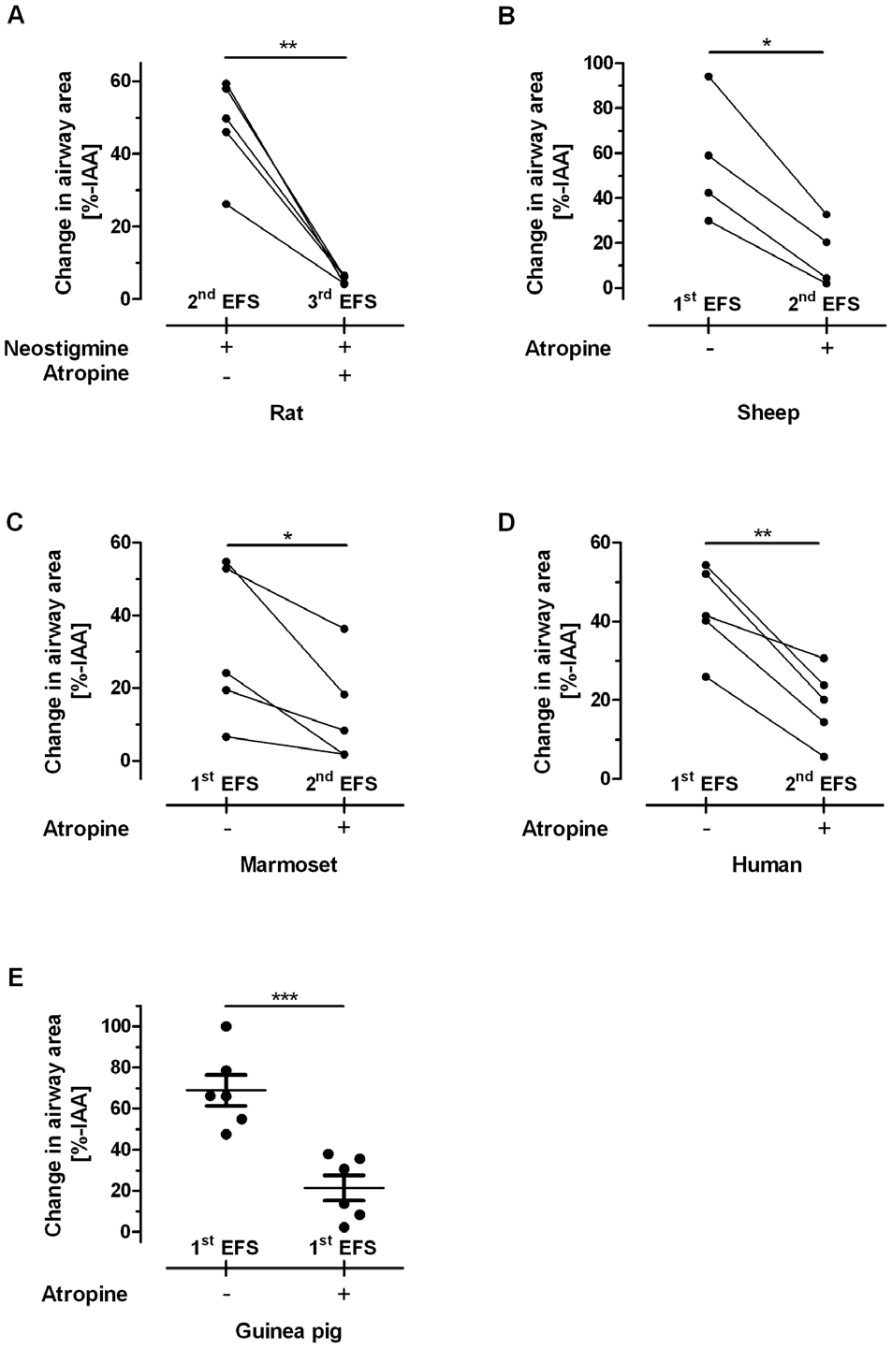
Cholinergic responses in EFS-triggered airway contraction. The PCLS from either rats (n = 5) (**A**), sheep (n = 4) (**B**), marmosets (n = 5) (**C**) or human (n = 4) (**D**) were stimulated repeatedly at F = 50 Hz, B = 1 ms, A = 200 mA, TW = 2.5 s, TR = 60 s for 3.3 min, i.e. 4×EFS. As control, the first stimulation (1^st^ EFS) was always carried out without any cholinergic interference. In subsequent EFS separated by at least 15 min to the preceding stimulation, neostigmine and/or atropine were added to augment or inhibit cholinergic airway contraction. The response in each single PCLS before and after treatment is plotted and matched to each other by a line *, p<0.05 and **, p<0.01 for atropine *vs.* no atropine (paired t-test). **E.** To prove cholinergic responses in guinea pig (n = 6), PCLS were electrically stimulated once (F = 50 Hz, B = 1 ms, A = 200 mA, TW = 2.5 s) either after pre-incubation with atropine or without interference. The mean with SEM is indicated by the horizontal line and the error bars, respectively. ***, p<0.001 in unpaired t-test.

Having proven the neural origin of EFS-induced airway contraction in PCLS, the types of nerves inducing bronchoconstriction were determined. The muscarinic antagonist atropine was used to address parasympathetic cholinergic innervation. Atropine significantly reduced airway contractions following repeated EFS of rat, sheep, marmoset and human PCLS (figure 4 A–D). Of note, in the case of rat PCLS the acetylcholine esterase inhibitor neostigimine was used to augment the response, because initial responses were only moderate and insufficient to show significance. In guinea pigs, PCLS had to be pre-incubated with atropine before the first stimulation in order to demonstrate cholinergic airway innervation (figure 4 E), since reversible stimulation was impossible due to an incomplete relaxation to the IAA.

**Figure 5:**
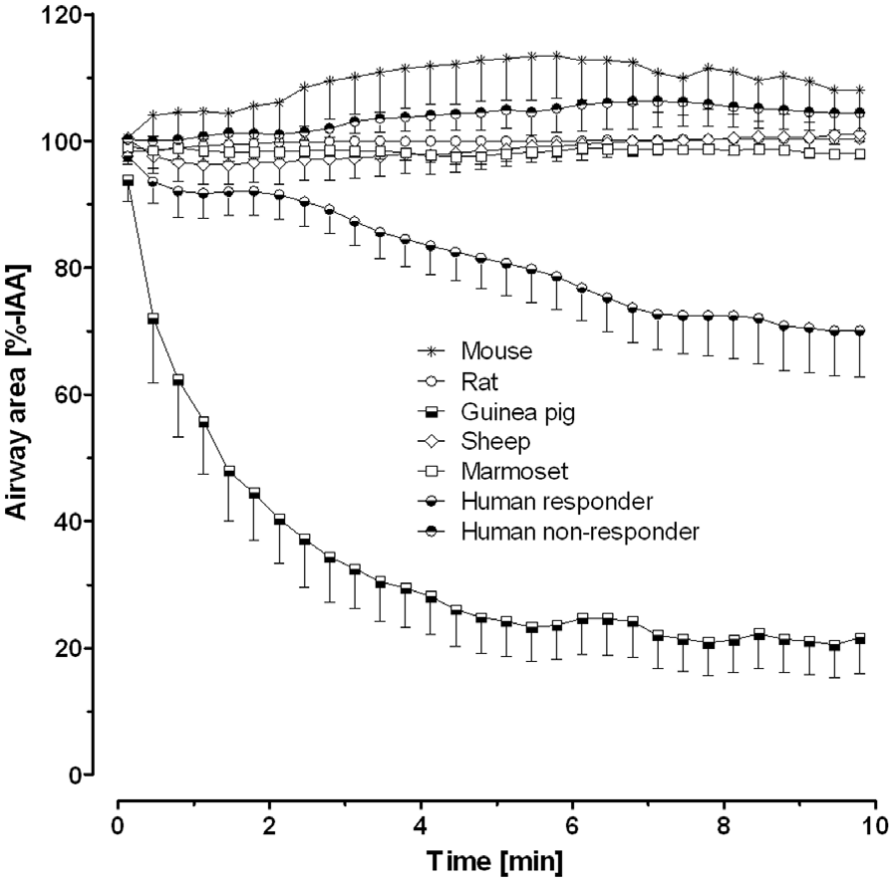
Effect of capsaicin on airway tone in precision-cut lung slices (PCLS). Capsaicin was applied to PCLS from the indicated species at a concentration of 10 µM and airway contraction was monitored. Data are shown as mean – SEM (species/number of PCLS/number of subjects: mouse/6/6, rat/10/4, guinea pig/9/9, sheep/4/4, marmoset/3/3, human responder/6/4 and human non-responder/5/3.

Neurally triggered airway contraction may not be restricted to cholinergic innervation, but can also be due to eNANC innervation. Therefore, we studied the effect of the TRPV1 agonist capsaicin, which leads to the tachykinin release from eNANC nerves [31]. Capsaicin induced strong airway contractions in guinea pigs and modest contractions in human (figure 5); of note, however, in three out of seven human lungs none of the studied PCLS responded to capsaicin. In mouse, rat, sheep and marmoset capsaicin did not contract airways (figure 5).

**Figure 6:**
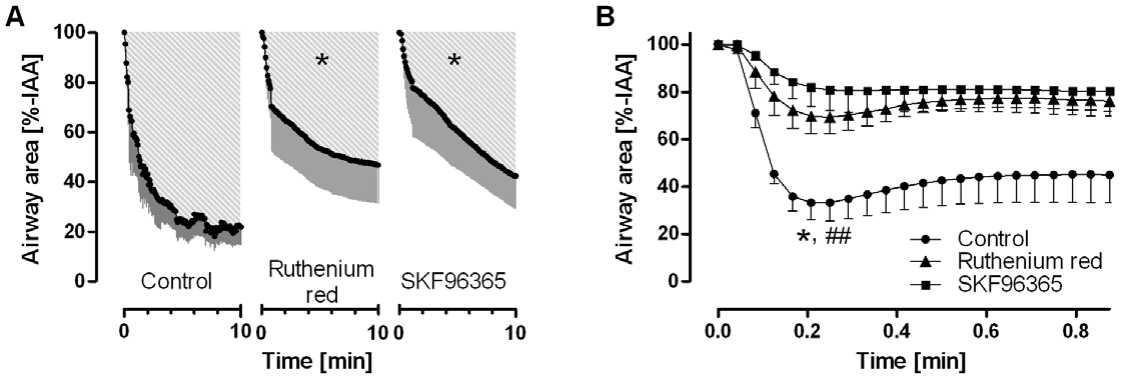
Characterization of peripheral eNANC airway responses in guinea pigs. **A.** Capsaicin was added to precision-cut lung slices (PCLS) at a concentration of 10 µM, either after 15 min pre-incubation with an unspecific transient receptor potential (TRP) channel antagonist (ruthenium red, SKF96365) or without pre-incubation (control). Data are shown as mean – SEM; n = 5 PCLS from 5 guinea pigs. 100% of the initial airway area (IAA) was considered as baseline and the area above the curve (AAC), i.e., the area enclosed by the baseline and the curve, is depicted as shading. Statistical analysis was performed on the AAC; *, p<0.05 in ANOVA followed by the Tukey test. **B.** Electric field stimulation (EFS; F = 50 Hz, B = 1 ms, A = 200 mA, SD = 2.5 s) was carried out on PCLS either after pre-incubation with TRP channel blockers or without pre-treatment (control). The minimal airway area obtained in the EFS of PCLS was used in statistical analysis showing an effect for both TRP channel antagonists. Data are shown as mean - SEM; n = 6 PCLS from 6 guinea pigs; *, p<0.05 and ^##^, p<0.01 for the comparison of the control group to the ruthenium red and SKF96365 group, respectively, in the Tukey multiple comparison test.

To further characterize the strong response to capsaicin in guinea pigs and to test whether TRP channels are pharmacologically accessible in PCLS, PCLS were incubated with the unspecific TRP channel blockers SKF96365 or with ruthenium red prior to stimulation by capsaicin. Both inhibitors significantly reduced the response to capsaicin (figure 6 A).

The observation that in guinea pigs airways did not relax back to the IAA after an EFS train (figure 1), even if recovery periods were prolonged (data not shown), suggested that a non-cholinergic mechanism may be involved. This was studied by the application of ruthenium red and SKF96365 during EFS of guinea pig PCLS. As PCLS could not be stimulated repeatedly due to the lack of relaxation, PCLS were again grouped and incubated with the inhibitors prior to the first stimulation. Ruthenium red and SKF96365 (figure 6 B) were both effective in blocking the response compared to the EFS only group.

## Discussion

In the present study an electric field was applied to PCLS from different mammals allowing the comparison of neuronally triggered airway responses under identical conditions. Species were heterogeneous in their response: In rats, sheep, guinea pigs, marmosets and humans bronchoconstriction was caused by cholinergic activation. Only in PCLS from guinea pigs and humans did activation of eNANC nerves contribute to the airway contractions.

### General considerations on EFS of PCLS

Recently, we introduced rat EFS as a means to study neural responses in PCLS and found the following conditions to be effective [21]: F = 50 Hz, B = 1 ms, A = 200 mA and TW = 2.5 s. The same parameters were used to stimulate PCLS in the present study and evoked characteristic airway contractions in each species (figure 1), except for the mouse. The neural mediation of these responses was demonstrated by the inhibitory effect of magnesium concentrations (10 mM) high enough to prevent voltage-induced calcium entry and the release of neurotransmitters at the nerve terminals [27], [28] (table 1). We showed that the contraction to exogenous methacholine was not affected by magnesium to exclude that the high magnesium concentration alters ASM physiology itself. These findings show that under our conditions EFS acts on neurons and not directly on ASM.

Under our standard conditions (F = 50 Hz, B = 1 ms, A = 200 mA≙40 V, TW = 2.5 s) mice were completely unresponsive. However, EFS elicited contractions in tracheal preparations of mice in previous studies [32], and in line with that we observed neurally evoked bronchoconstriction in murine PCLS at higher electric fields (figure 2). In sheep PCLS, on the other hand, airways were highly excitable (figures 1, 3) and nerve activation was observed already at low frequencies or even after one single electrical stimulus (0.4 Hz at train width 2.5 s). Overall, the EF_50_ values that we observed are in line with prior studies on bronchial or tracheal preparations [33]–[36], which demonstrates that PCLS are a suitable tool to study neural regulation of airway tone. Among all species studied here, the EF_50_ value of guinea pig PCLS (7.0±0.5 Hz) is closest to that of humans (10.2±0.4 Hz), providing an argument for our conclusion that guinea pig airways are a reasonable proxy for human airways.

We believe that the differences between the species are not explained by differences in ASM cells, because the responses to methacholine are very similar in all species [10], [12], [13], [15], [16], [18]. Additionally, the experiments with magnesium exclude direct electric activation of ASM. Possible explanations are differences in neurotransmitter release or degrading enzymes such as the acetylcholine esterase that may vary in amount or activity [37]–[39]. However, we believe that the type of innervation, i.e., cholinergic, adrenergic, eNANC or iNANC, is the most relevant reason for the differences between species, as for instance the repetitive reversible contraction in rat is cholinergic [21], whereas the steady contraction without relaxation in guinea pigs (figure 1) suggests an additional mechanism other than a cholinergic one in which the neurotransmitter is not metabolized.

### Pharmacological characterization of distal neural airway responses

The type of innervation was addressed by pharmacological agents: The muscarinic antagonist atropine was used to show the involvement of parasympathetic cholinergic nerves. The TRPV1 channel activator capsaicin as well as the unspecific TRP channel blockers SKF96365 or ruthenium red were used to address eNANC nerves.

Atropine blocked the EFS-induced bronchoconstriction in PCLS from rats, guinea pigs, sheep, marmosets and humans (figure 4), indicating that these species receive cholinergic innervation in the distal parts of their lungs. These findings extend former studies on larger airways [3], [33] and provide important new information, because innervation is unevenly distributed along the tracheobronchial tree [33]. This is also true for tachykinergic innervation in guinea pigs that before was thought to supply predominantly the large airways [33]. In this species, distal airways appear to be supplied by both cholinergic and tachykinergic nerves, as shown by the effects of atropine and the TRP channel blockers (figure 4 and 6). Moreover, in contrast to the rats, in which cholinergic responses are weaker in smaller airways [21], distal airways from sheep or human were quite susceptible to EFS-induced cholinergic bronchoconstriction (figure 4), indicating relatively strong innervation of peripheral airways in these species. The residual airway contractions in the presence of atropine may be due to eNANC activation, due to blockade of presynaptic muscarinic receptors by atropine, preventing the autoinhibitory effect of acetylcholine release [40], [41] or due to release of substance P that might potentiate the release of acetylcholine from cholinergic nerves [40].

Various types of TRP channels are expressed in the mammalian lung and are involved in the regulation of airway functions, in which particularly TRPV1 and TRPV4 play an important role. TRPV1 channels are generally expressed in non-myelinated C-fiber afferents, which innervate the airways and contain sensory neuropeptides [42]. Activation of TRPV1 channels on nerves induces calcium influx into neurons, membrane depolarization and action potentials. Such excited chemosensitive nerves correspond to eNANC nerves, which induce bronchoconstriction by the release of substance P or a related tachykinin [43]. Therefore, we studied the effects of capsaicin, a potent TRPV1 agonist [31], [42], to examine the role of eNANC innervation in the various species. Notably, only airways of guinea pigs and human responded to capsaicin providing further evidence for the presence of tachykinergic nerves in the distal lungs of these species (figure 5) and further suggesting the guinea pig as a good model for the human situation. In support of this conclusion, Undem and coworkers [29] reported contractions of 66±10% in guinea pig bronchi towards capsaicin and Spina's group [3] made similar observations in human airways (1 µM: 2±3%, 10 µM: 14±13% and with 100 µM capsaicin 44±40% of the maximal contraction by 10^−4^ M methacholine). Studies by Lundberg and colleagues [44] support the notion that human airways are less responsive to capsaicin than those from guinea pigs, by showing guinea pig tracheas being 1000-fold more sensitive to substance P and 100-fold more sensitive to capsaicin than human bronchi. Further, Lundberg's and colleagues' [44] study also supports our observation that not all human airways are capsaicin sensitive. From studies with transmural EFS of human segmental bronchi, they report two different scenarios: one where contractions were abolished by atropine and another one where only tetrodotoxin but not atropine was effective. These findings suggest the existence of two types of human airways: either solely innervated by cholinergic nerves or innervated by both cholinergic and eNANC nerves.

That capsaicin failed to evoke clear bronchoconstriction in PCLS from marmoset is in line with data from marmoset tracheae and bronchi [3]. These only contracted by 100 µM capsaicin up to 2.3±2.8% respectively 8.0±6.6% of the maximal contraction induced by 100 µM methacholine [3]. In rat and mouse bronchi, but probably also in marmoset and sheep airways, capsaicin may play a more prominent role in the activation of sensory nerves of the inhibitory system than in the excitatory system [45], [46]. This is especially true for the mouse, because in mouse PCLS the airway relaxed in response to capsaicin by 13±6.7% (figure 5). As contraction in sheep airways are not completely reversible under standard condition (figure 1) a capsaicin response would be expected. However, lack of full reversibility is due to incomplete recovery phase between electric stimulation. Prolonged recovery phase leads to full reversibility of sheep airways. In contrast to rat airways, the full reversibility of sheep airways within in one minute is not achieved, since first contraction of sheep airways was much stronger and second the concentration or activity of the acetylcholine esterase in the synaptic cleft may have been low. Latter argument is supported by our unpublished observation, that neostigmine fails to increase EFS-induced bronchoconstriction in sheep (mean of contractions±SEM: 50.4±12.5%-IAA w/o neostigmine vs. 71.3±5.4%-IAA plus neostigmine, n = 5, p = 0.1594 in t-test).

Neural stimulation can also cause bronchorelaxation via either sympathetic or iNANC nerves [2]. In the present study, only marmoset airways enlarged to a caliber greater than the initial caliber after multiple EFS (figure 1). Only the combination of a β-receptor antagonist (propranolol) and a nitric oxide synthase inhibitor (L-NAME) was effective to block the relaxation (data not shown) suggesting that both types of nerves are involved in regulating marmoset airway tone. Hence, the marmoset may be a useful model to study relaxant innervation of the lung, especially as no precontracting agents are needed. Whether the peripheral airways of the other species are or are not innervated by relaxant nerves has still to be elucidated, because usually relaxation becomes apparent only in precontracted airways [47].

Airway pharmacology varies strikingly between species, not only with respect to neural transmission, but also with respect to various inflammatory mediators. For instance, airways from rodents, marmosets or rhesus monkeys do respond weakly or not at all to leukotrienes [16], [48], that readily contract airways in humans [49], cynomologus, baboons [16] and guinea pigs [13]. The ready contraction of guinea pig airways by leukotrienes is not only found in PCLS, but also in tracheal or parenchymal preparations as well as *in vivo* [50]. In contrast, in ventilated mice leukotrienes did not induce bronchoconstriction [51]. Therefore from our observations and from the literature two conclusions can be made: first species differences observed in PCLS translate to other model systems and most likely to *in vivo*; second, with respect to airway pharmacology guinea pigs resemble humans more closely than do mice or rats [52], [53]. Belonging to the family of callitrichidae, the non-human primate marmoset is the closest relative to humans among the species studied [54]. Neural airway responses were similar between the marmoset and humans and we therefore consider the marmoset as a useful primate model to study airway pharmacology.

In conclusion, for the first time airway innervation was studied in various mammals including humans in the same lab and under identical conditions. PCLS represent a useful model to examine peripheral airway innervation, because nerves remain functional and can be activated specifically. Using this model we showed that the small airways of rats, guinea pigs, sheep, marmosets and human do all receive cholinergic innervations. Moreover, distal guinea pig and human airways do also receive eNANC innervations. Furthermore, it was shown that PCLS are a suitable tool to investigate physiological consequences of TRP channels in the lung. Since guinea pig and marmoset neural responses correlate with the human responses reasonably well, we consider these species suitable for studies on the role of peripheral airway innervations relevant to human lung diseases.
